# Genomic Analysis of the First European Bacteriophages with Depolymerase Activity and Biocontrol Efficacy against the Phytopathogen *Ralstonia solanacearum*

**DOI:** 10.3390/v13122539

**Published:** 2021-12-17

**Authors:** Elena G. Biosca, José Francisco Català-Senent, Àngela Figàs-Segura, Edson Bertolini, María M. López, Belén Álvarez

**Affiliations:** 1 Departamento de Microbiología y Ecología, Universitat de València (UV), 46100 Valencia, Spain; jfcatala@cipf.es (J.F.C.-S.); angela.figas@uv.es (À.F.-S.); edson.bertolini@ufrgs.br (E.B.); mariabelen.alvarez@madrid.org (B.Á.); 2 Centro de Investigación Príncipe Felipe, Unidad de Bioinformática y Bioestadística, 46012 Valencia, Spain; 3 Faculdade de Agronomia, Universidade Federal do Rio Grande do Sul (UFRGS), Porto Alegre 91540-000, Brazil; 4 Centro de Protección Vegetal y Biotecnología, Instituto Valenciano de Investigaciones Agrarias (IVIA), 46113 Valencia, Spain; mm52lopez@gmail.com; 5 Departamento de Investigación Aplicada y Extensión Agraria, Instituto Madrileño de Investigación y Desarrollo Rural, Agrario y Alimentario (IMIDRA), 28800 Alcalá de Henares, Spain

**Keywords:** bacterial wilt, biological control, phage, microscopy, sequencing, molecular characterization, genomic characterization, depolymerase

## Abstract

*Ralstonia solanacearum* is the causative agent of bacterial wilt, one of the most destructive plant diseases. While chemical control has an environmental impact, biological control strategies can allow sustainable agrosystems. Three lytic bacteriophages (phages) of *R. solanacearum* with biocontrol capacity in environmental water and plants were isolated from river water in Europe but not fully analysed, their genomic characterization being fundamental to understand their biology. In this work, the phage genomes were sequenced and subjected to bioinformatic analysis. The morphology was also observed by electron microscopy. Phylogenetic analyses were performed with a selection of phages able to infect *R. solanacearum* and the closely related phytopathogenic species *R. pseudosolanacearum*. The results indicated that the genomes of vRsoP-WF2, vRsoP-WM2 and vRsoP-WR2 range from 40,688 to 41,158 bp with almost 59% GC-contents, 52 ORFs in vRsoP-WF2 and vRsoP-WM2, and 53 in vRsoP-WR2 but, with only 22 or 23 predicted proteins with functional homologs in databases. Among them, two lysins and one exopolysaccharide (EPS) depolymerase, this type of depolymerase being identified in *R. solanacearum* phages for the first time. These three European phages belong to the same novel species within the *Gyeongsanvirus*, *Autographiviridae* family (formerly *Podoviridae*). These genomic data will contribute to a better understanding of the abilities of these phages to damage host cells and, consequently, to an improvement in the biological control of *R. solanacearum*.

## 1. Introduction

*Ralstonia solanacearum* is a primary plant pathogen responsible for bacterial wilt, one of the most destructive and widespread plant diseases [1,2]. This soil and water-borne Gram-negative bacterium affects economically important solanaceous crops as well as many ornamental plants, thus being a major threat to agriculture worldwide [1,3,4,5,6]. Since the first detection in Europe in 1972 [7], bacterial wilt outbreaks have been reported in Northern, Western and Mediterranean European countries, this pathogen being included in the Priority Pest List of Pests of Economic and Environmental Importance in the European Union (EU) [6]. 

The bacterium has long been part of the “*R. solanacearum* species complex” (RSSC) composed of strains with high variability. In 2005, RSSC strains were classified in four phylotypes (I–IV), correlated with different geographic origins [8], although these phylotypes are likely to be disseminated worldwide through infected plant material [9]. After outbreaks in the EU, RSSC strains isolated from potato, tomato, soil, waterways and weeds were classified as phylotype II [3,9,10,11,12,13,14,15]. In 2014, the RSSC experienced a major taxonomic revision [16] with only phylotype II strains being classified as the current *R. solanacearum* species, present in the EU [6,17] and many countries of America [17], the continent where the pathogen was first described [18]. 

Currently, there are no effective *R. solanacearum* control methods [19]. In the search for new treatments, while agrochemicals have an impact in the ecological balance and human health, most biocontrol strategies are environmentally friendly and easy to integrate in a sustainable agricultural system. Among them, those based on bacteriophages (phages) have great potential [20,21]. Phage-based biocontrol of plant diseases has been focused on the action of lytic phages because of their numerous advantages [20,22,23]. Phages active against bacterial species close to *R. solanacearum* such as *R. pseudosolanacearum* and/or *R. syzygii* subsp. *indonesiensis* have been reported [4] but, until very recently, no phage was described to have successful biocontrol potential against *R. solanacearum in planta* and in environmental water. An innovative biocontrol methodology for bacterial wilt prevention and/or control based on the activity of new three lytic phages specific against the present *R. solanacearum* species, named vRsoP-WF2, vRsoP-WM2, and vRsoP-WR2, was reported [5] and patented [24,25,26]. These *R. solanacearum* phages are effective in reducing both high populations of the pathogen in environmental water and bacterial wilt incidence *in planta* [5,24,25,26]. They were initially characterized [5,24,25,26] but a deeper genetic analysis of their genomes was needed to understand the biology of these waterborne phages and increase the knowledge on their biocontrol activity. 

In this study, the morphological and genomic characteristics and taxonomic classification of the first European phages with biocontrol efficiency against the present species *R. solanacearum* are described. The results have revealed that they are closely related and members of the same novel species within the genus *Gyeongsanvirus* and the *Autographiviridae* family. Their genomic analysis further confirmed their lytic lifestyle and identified two lysins and a new type of exopolysaccharide (EPS) depolymerase in *R. solanacearum* phages, proteins that are essential components to damage their host cells. This new information has confirmed their suitability as safe biological control agents and has shed light on their biocontrol abilities on *R. solanacearum*.

## 2. Materials and Methods

### 2.1. Bacterium, Phages, and Growth Conditions

The strain CFBP 4944 (or DSMZ 100387) of *R. solanacearum* isolated from potatoes with brown rot symptoms in Spain [5,11] was the bacterial host in all phage assays. It was routinely cultured on the general media casamino acids peptone glucose (CPG) [27] or Luria Bertani (LB) [28] with 1.5% bacteriological agar (CPGA or LBA), for 48 h at 28 °C. LB broth (LBB) was used for overnight cultures of the bacterial strain at 28 °C with aeration by shaking at 120 rpm.

Phages vRsoP-WF2, vRsoP-WM2 and vRsoP-WR2 were isolated from different rivers geographically far distant in Spain in 2001, 2003 and 2004, respectively, and showed lytic activity and biocontrol efficacy against *R. solanacearum* [5,24,25,26], were further characterized in this work. They were propagated on *R. solanacearum* cultures similarly to Álvarez et al. [5] with minor modifications. Briefly, the host strain was grown in LBB overnight at 28 °C and 120 rpm. Thereafter, bacterial cell concentration was adjusted to an OD_600 nm_ = 0.5, equivalent to about 10^8^ colony forming units (CFU)/mL using a spectrophotometer (Thermo Scientific Genesys 20, Waltham, MA, USA). Afterwards, 0.1 mL of 0.22-μm-filtered lysates were added to 5 mL of adjusted bacterial suspensions and the mixture was incubated overnight at 28 °C and 120 rpm. Phage titers were determined by performing serial 10-fold dilutions of each phage lysate (0.22 μm-filtered lysates) in SM buffer [29] and the double-layer agar method. Thus, 0.2 mL aliquots of the bacterial culture (adjusted to an OD_600 nm_ = 0.5) were mixed with 0.1 mL of each phage dilution and 5 mL of soft agar (0.6%) medium, poured onto CPGA plates, and incubated at 28 °C for 48 h. After incubation, the plaque forming units were observed. Phage suspensions were maintained for short periods at 4 °C.

### 2.2. Phage Lytic and Depolymerase Activities

Lytic and depolymerase activities of the three phages were determined by the double-layer agar plate method used for phage titration as mentioned above, and onto culture media previously inoculated with *R. solanacearum* according to a standard surface plating method [5]. After determining the PFU/mL by counting clear plaques (lytic zones), plates were incubated and observed during several days to detect the depolymerase activity as the appearance of a turbid halo surrounding the initial lytic zone. This activity was determined using CPGA, LBA, Yeast Extract Peptone Glucose Agar (YPGA) [30] and King’s medium B (KB) [31] plus 1.5% agar (KBA) culture media for 48–72 h at 28 °C and up to 7 days at 4 °C.

### 2.3. Phage Morphology

Phage virions were prepared from 0.22 μm-filtered lysates obtained as described above. Thereafter, 5 μL of these lysates were separately absorbed on fresh formvar and carbon covered grids for 1 min. Excess of samples was removed and the grids were stained with 1% of phosphotungstic acid (PTA) (pH 7.0) for 1 min and air-dried. Electron microscopic visualizations of the phage virions were performed using the JEM-1010 (JEOL) transmission electron microscope (TEM) operated at 80 kV and with 8 Mpx AMT digital camera from the Central Service for Experimental Research (SCSIE) at Universitat de València. Virion dimensions were determined on micrographs with TEM software, with at least 15 different virions.

### 2.4. Phage DNA Isolation and Genome Sequencing

DNA was isolated from the virions of the three phages similarly to Álvarez et al. [5], but using a commercial kit. Briefly, bacterial nucleic acids were degraded by treating filtered phage lysates and precipitated samples with DNase and RNase for 1 h at 37 °C, which were later heat inactivated. Thereafter, phage DNA was isolated by using the kit for the isolation of low-copy plasmids of NucleoSpin^®®^ Plasmid (Macherey-Nagel, Düren, Germany). DNA concentration and quality were determined spectrophotometrically at 260 nm and 280 nm with a Nanodrop (ND-2000, Thermo Fisher, Wilmington, DE, USA).

DNA libraries were constructed with Nextera XT Library Preparation Kit (Illumina, San Diego, CA, USA) for the three phage genomes and the sequencing process was carried out using the Illumina MiSeq platform with 2 × 250 bp paired end sequencing (Illumina, USA). The resulting reads were filtered and trimmed with Trimmomatic v0.36 [32]. 

### 2.5. Phage Genome Bioinformatic Analysis

#### 2.5.1. De Novo Assembly

De novo assembly of the filtered sequences was performed using Unicycler v0.4.9b [33]. Briefly, this bioinformatics pipeline used SPAdes v3.14.1 [34] for read assembly, and Pilon v1.23 [35] for correction of small errors in the assembly, if detected. Phage termini were confirmed by Sanger sequencing and supported by phylogenetic inference of large DNA terminase protein sequences. Prediction of lytic or lysogenic lifecycle was performed with the tool PhageAI v0.11 (www.phage.ai accessed on 24 October 2021).

#### 2.5.2. Genome Annotation

The prediction of the open reading frames (ORFs) was carried out with RASTtk [36], using Glimmer3 [37] and Prodigal [38] for gene annotation. The predicted ORFs were reviewed and refined through database searches using the BLASTp [39] and HHpred [40] online tools. Virfam was additionally used for the detection of the proteins of the head-neck-tail modules [41]. The functional annotation results generated by RASTtk were complemented with previous BLASTp and HHpred searches. In addition, a search for conserved domains and important sites in proteins was performed with InterProScan [42], and a prediction of transmembrane helices (TMH) was carried out with the software TMHMM V.2.0 [43]. The absence of virulence factors and antibiotic resistance genes was determined by performing a PathoFact analysis [44] and a Blast against the VFDB (virulence factor database) [45], respectively.

#### 2.5.3. Phylogenetic Analysis

Phylogenetic analysis was performed with the whole genomes of the three river water European phages and a selection of their 26 closest viruses belonging to the *Autographiviridae* family (formerly *Podoviridae*) able to infect either *R. solanacearum* (three African phages [46,47], one American [48] and one Asian (accession number: MF979559) or the closely related species *R. pseudosolanacearum* (21 phages from diseased plants, soil or irrigation water isolated from Africa (14) [49] and Asia (7) [50,51,52]). 

A genome-based phylogeny was generated using VICTOR [53], intergenomic distances were calculated with the Genome BLAST Distance Phylogeny (GBDP) d0 formula for nucleotidic sequences. Intergenomic distances/similarities were also calculated with VIRIDIC [54], which implements the algorithm used by the International Committee on Taxonomy of Viruses (ICTV). Both VICTOR and VIRIDIC are able to group the analysed sequences within genus and species according to the calculated distances. A proteomic tree was constructed from the viral genomes with the ViPTree web server [55] based on normalised tBLASTx scores. The packaging strategy of the *R. solanacearum* European phages was predicted according to Casjens and Gilcrease [56]. Briefly, the terminase large subunit genes from the three European phages of *R. solanacearum*, their four closest phages and 50 reference phages [57] (Table S2) were aligned at the amino acid level using ClustalW in accurate mode (www.genome.jp/tools-bin/clustalw accessed on 25 October 2021). PhyML with Smart model Selection [58,59] was used with the Akaike information criterion (AIC) together with the fast likelihood-based method aLRT SH-like to generate a ML tree. The tree was generated using the iTol online tool [60].

## 3. Results

### 3.1. Phage Plaque Morphology, Lytic and Depolymerase Activities

**Figure 1:**
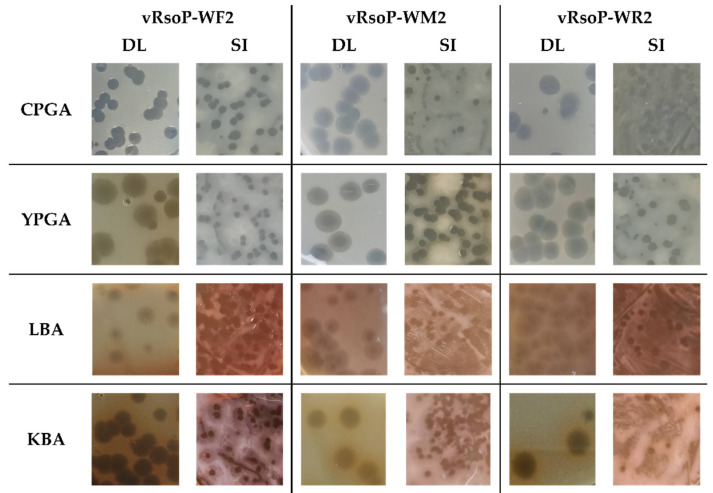
Morphology of vRsoP-WF2, vRsoP-WM2 and vRsoP-WR2 plaques. The phages produced clear plaques surrounded by translucent halos after prolonged incubation with strain CFBP 4944 of *R. solanacearum* in CPGA, YPGA, LBA and KBA culture media. Clear plaques due to lytic activity were observed after 24 h at 28 °C and the translucent halos around the lytic plaques were increasing in size within time after 2–3 days at 28 °C, and 7 days at 4 °C in double layer (DL) and surface inoculation (SI) methods.

The plaque morphology of each phage after incubation with *R. solanacearum* strain CFBP 4944 at 28 °C 24–48 h was a transparent halo due to lytic activity, which was observed on LBA, CPGA, YPGA and KBA plates, inoculated both by the double-layer agar plate method and by the standard surface plating method. However, prolonged incubation for 2–3 days allowed the observation of translucent halos surrounding the plaques of the phages on the different culture media assayed. These halos that expand over time suggest the production of enzymes able to degrade the EPS of the host bacteria and therefore depolymerase activity (Figure 1). The detection of the depolymerase activity was possible in all conditions assayed but, it was better observed on YPGA, followed by CPGA and using the double layer method. Besides, *R. solanacearum* produced a brownish pigment on KBA and LBA media, turning the culture medium into a brownish dark colour, which made the depolymerase activity detection more difficult than on YPGA and CPGA, both of them containing glucose (Figure 1). 

### 3.2. Phage Virion Morphology

**Figure 2:**
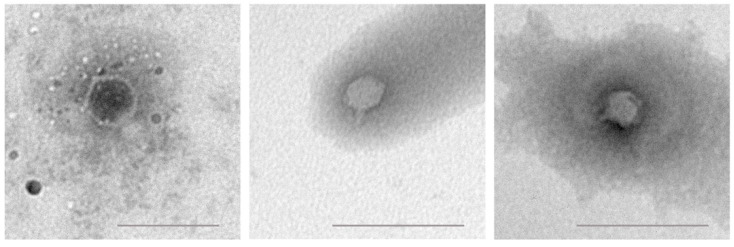
Virion morphology of vRsoP-WF2 (**left**), vRsoP-WM2 (**center**), and vRsoP-WR2 (**right**). Transmission electron micrographs of negatively stained *R. solanacearum* virions magnified at ×30,000 (vRsoP-WF2), and ×20,000 (vRsoP-WM2 and vRsoP-WR2). Scale bars represent 100 nm in vRsoP-WF2, and 200 nm in vRsoP-WM2 and vRsoP-WR2 micrographs, respectively.

Virion visualization by transmission electron microscopy indicated that phages vRsoP-WF2, vRsoP-WM2 and vRsoP-WR2 may belong to the *Autographiviridae* family (formerly *Podoviridae*), since they showed icosahedral heads of about 50 nm in diameter and short non-contractile tails from about 19–26 nm in length (Figure 2). For the vRsoP-WF2, heads showed an average size of 49.75 ± 2.45 nm and tail size was estimated at about 25.57 ± 4.3 nm. In the case of vRsoP-WM2, heads were of 48.87 ± 4.87 nm and tails around 19.68 ± 5.47 nm. vRsoP-WR2 showed an average head size of 50.22 ± 2.91 nm and tail size was estimated around 19.36 ± 5.28 nm.

### 3.3. Phage Genomic Analysis

The assembly of the phage sequences resulted in three complete double-stranded DNA genomes with sizes of 40,690 bp for vRsoP-WF2, 40,688 bp for vRsoP-WM2 and 41,158 bp for vRsoP-WR2 (Table 1). The different genome size between the last phage and the other two was due to a large insertion of 468 bp in vRsoP-WR2. The GC-contents in the three phages ranged from 59.04% to 59.10% (Table 1). PhageAI classified vRsoP-WF2, vRsoP-WM2 and vRsoP-WR2 as virulent or lytic phages, with percentages of 85.98, 84.15 and 86.12, respectively. Protein annotation with RASTtk and its subsequent refinement resulted in the detection of 52 ORFs in vRsoP-WF2 and vRsoP-WM2, and 53 in vRsoP-WR2 (Table 1).

**Figure 3:**
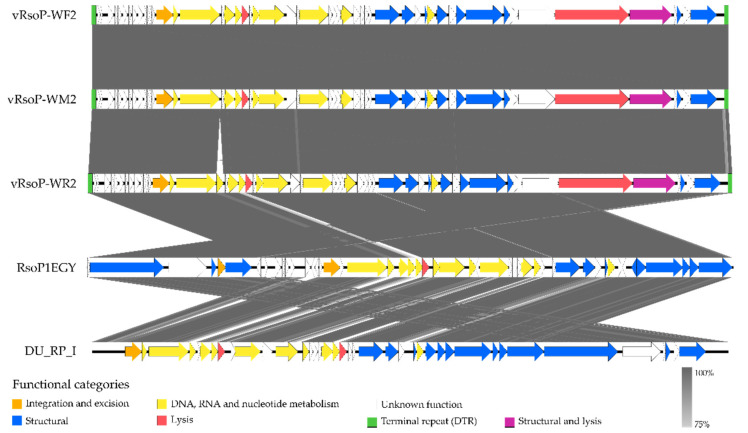
Genomic organization of the three European *R. solanacearum* phages together with the closest phages RsoP1EGY and DU_RP_I. Open reading frames (ORFs) are shown using arrow symbol, which indicates the direction of transcription. Genes grouped in different functional categories are shown in different colours according to the legend below the map. The categories of the phage proteins of RsoP1EGY and DU_RP_I were extracted from the PHROG database [61]. The genomic map was generated with the genome comparison visualiser EasyFig 2.2.5 [62].

The additional protein in vRsoP-WR2 was found in the large insertion, as shown in Figure 3. The accession number of the genome sequences of the three phages deposited in the GenBank database and a summary of their characteristics are shown in Table 1. Using RASTtk and InterProScan tools, only 22 or 23 out of 52 or 53 ORFs were identified to share similarities with previously reported genes in the database, showing homology to functionally characterized genes. These ORFs and their putative functions are shown in Table 2, and the ORF positions and their protein translations in Table S1. No ORFs associated with virulence or antibiotic resistance were identified by using PhatoFact and VFDB, respectively. The European *R. solanacearum* phage genomes were arranged in several functional modules, characteristics of the *Autographiviridae* family: A first one for early transcribed genes encoding for many hypothetical proteins with unknown function, a second one including genes for DNA metabolism, and a third one with genes encoding for structural proteins. No lysis cassette was identified as such, although genes for host lysis were annotated (Figure 3). Direct terminal repeats (DTR) were found at the ends of the genomes, as described below. The size of these DTRs was 281 bp for vRsoP-WF2 and vRsoP-WR2, and 280 bp for vRsoP-WM2.

The module for early transcribed genes encoded for many hypothetical proteins with no known functional or structural domain except for ORF 14, which was predicted to be a putative integrase/tyr recombinase in the three phages (Figure 3, Table 2). ORF 14 shared homology to other integrases found in close related lytic *R. solanacearum* or *Ralstonia* sp. phages like RsoP1EGY, PSG-11, PSG-11-1, and unrelated lytic phages such as *Bordetella bronchiseptica* phage vB_BbrP_BB8 [63]. However, no lysogeny modules were detected either in the genomes of the three phages, nor in the genomes of the other lytic phages mentioned above.

Nine ORFs were predicted to be involved in DNA metabolism in two of the European *R. solanacearum* phages, with an additional ORF annotated in vRsoP-WR2 (Figure 3, Table 2). Eight or nine of them were required for DNA replication and modification. Thus ORF 15 encoded for a putative HNH endonuclease in the three phages, which play important roles in the phage life cycle as a key component of phage DNA packaging [64]. An extra ORF present only in the large insertion of vRsoP-WR2 (ORF 17) was predicted to encode for a putative extra endonuclease also present in the most closely related *Ralstonia* phages, RsoP1EGY and DU_RP_I. This insertion at the nucleotide level shows 100% coverage with these two phages and high identity (99.8% for RsoP1EGY and 98.2% for DU_RP_I). No proteins or domains were identified in the ORF 17 of the other two phages. ORF 18 was predicted to be a single-stranded DNA-binding protein in vRsoP-WF2 and vRsoP-WM2, while this putative protein was encoded by ORF 19 in vRsoP-WR2. ORFs 19 and 22 coded for two additional putative endonucleases and ORF 39 for an endonuclease VII in vRsoP-WF2 and vRsoP-WM2; these putative proteins were identified in ORFs 20, 23 and 40 in vRsoP-WR2. ORFs 23 and 26 were predicted to be a DNA primase/helicase protein (sharing identity with Gp4A of phage T7) and a DNA-directed DNA polymerase, respectively, in vRsoP-WF2 and vRsoP-WM2; these proteins were identified in ORFs 24 and 27, respectively, in vRsoP-WR2. ORF 30 was annotated as an exonuclease in vRsoP-WF2 and vRsoP-WM2, while it was predicted by ORF 31 in vRsoP-WR2; this enzyme is necessary for molecular recombination and production of concatemers in phage T7 [65]. One ORF (ORF 16) was annotated to play a role in the transcriptional regulation in the three phages, a putative DNA-directed RNA polymerase. 

Ten ORFs were annotated to encode for phage structural proteins including capsid and tail-related putative proteins, as well as phage terminases (Figure 3, Table 2). Phage capsid-related proteins comprised several ORFs that were annotated as a collar, head-to-tail connector protein (ORF 35), a capsid assembly protein (ORF 36), a capsid and scaffold protein (ORF 38), a major capsid protein (ORF 40), and a probable scaffold protein (sharing identity with Gp13 of phage T7, IPR020335) (ORF 44) in vRsoP-WF2 and vRsoP-WM2; these putative proteins were encoded by ORFs 36, 37, 39, 41 and 45 in vRsoP-WR2. ORFs encoding phage tail-related proteins included ORF 42, annotated as a phage tail protein (tail tubular protein, sharing homology with Gp11 of phage T7), ORF 43 as a phage non-contractile tail tubular protein, and ORF 48, identified as a tail fiber protein (IPR005604, sharing identity with tail fiber of phage T7), respectively in vRsoP-WF2 and vRsoP-WM2; these proteins were encoded by ORFs 43, 44 and 49 in vRsoP-WR2. ORFs 50 and 52 were annotated as a terminase small subunit (Gp18) and a terminase large subunit (Gp19) in vRsoP-WF2 and vRsoP-WM2, and were predicted as ORFs 51 and 53 in vRsoP-WR2. These terminase proteins are associated with DNA packaging in phages. 

Three ORFs were annotated to be involved in cell lysis (Figure 3, Table 2). Two putative lysins were annotated in the three phages. One corresponds to ORF 20 in vRsoP-WF2 and vRsoP-WM2, and ORF 21 in vRsoP-WR2, encoding for a putative endolysin with a conserved amidase domain, involved in host lysis at the end of the lytic cycle for the release of phage progeny. This ORF exhibited high identity only with the nearest *Ralstonia* phages RsoP1EGY (100%), DU_RP_I (96.7%) and P-PSG_11 (89%). The other ORF (ORF 47 in vRsoP-WF2 and vRsoP-WM2, and ORF 48 in vRsoP-WR2) corresponds to a phage DNA-ejectosome component (Gp16, similar to that of phage T7), essential for phage morphogenesis and infection [66], a peptidoglycan lytic exotransglycosylase with a lysozyme-like domain (IPRO23346) found in glycosyl hydrolases and transglycosylases. No holin, to form pores in the inner membrane, or spanin, to disrupt the outer membrane in Gram-negative bacteria, were identified in the European phages, nor in the closest *Ralstonia* phages. However, by searching for domains of transmembrane helices in proteins by the TMHMM software, five ORFs (2, 33, 37, 44 and 49 in vRsoP-WF2 and vRsoP-WM2, and 2, 34, 38, 45 and 50 in vRsoP-WR2) were found that contain these transmembrane domains, whose presence has been described in holins and spanins [67,68].

**Figure 4:**
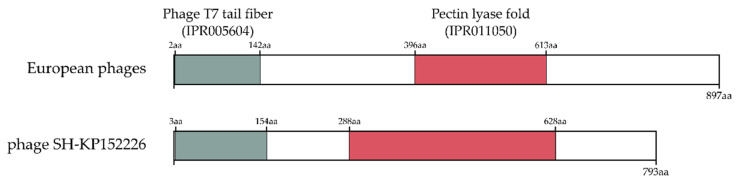
Comparison of tail fiber protein of the European *R. solanacearum* phages vRsoP-WF2, vRsoP-WM2 and vRsoP-WR2 (ORF 48 in vRsoP-WF2 and vRsoP-WM2, and ORF 49 in vRsoP-WR2) (coding for proteins of 897 aa) and the *K. pneumoniae* phage SH-KP152226 (ORF 42, coding for a protein of 793 aa). Grey and red regions correspond to phage T7 tail fiber (IPR005604) and pectin lyase fold/virulence (IPR011050) conserved domains, respectively.

An exopolysaccharide depolymerase was also identified in the ORFs coding for a tail fiber protein (ORF 48 in vRsoP-WF2 and vRsoP-WM2, and ORF 49 in vRsoP-WR2) in the three European viruses. Bioinformatic analysis of these ORFs showed that they encode for proteins with two conserved domains, a N-terminal T7-like tail fiber domain (IPR005604) and a central domain with a pectin lyase fold structure (IPR011050) (Figure 4). These two domains have a structure equivalent to that of the ORF 42 of the phage SH-KP152226 of *Klebsiella pneumoniae* [69], which conserved the amino acid (aa) sequence of the N-terminal domain (residues 2~142) and the central region with a domain (residues 396~613) identified with pectin lyase activity (IPR011050). No conserved domains were found in the C-terminal region (Figure 4). These ORFs exhibited high identity with the closest *Ralstonia* phages RsoP1EGY (99%), DU_RP_I (98%) and P-PSG-11 (97.77%), although RsoP1EGY and DU_RP_I have 85 fewer residues in the N-terminal region than the three European phages, while the size matches in the case of phage P-PSG-11.

### 3.4. Phage Classification

**Figure 5:**
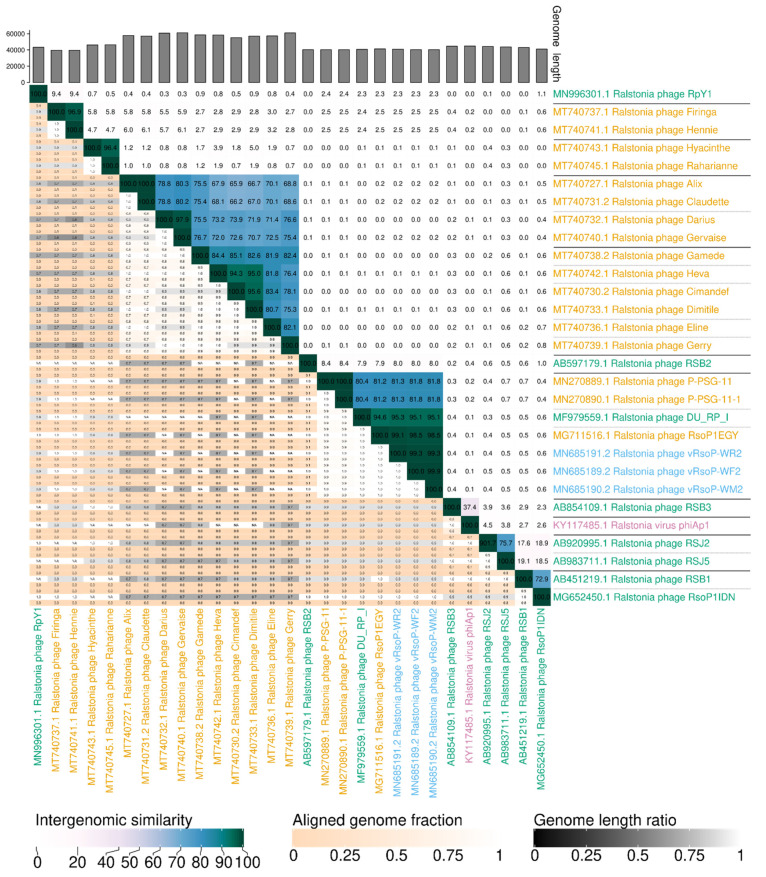
VIRIDIC generated heatmap of European water phages vRsoP-WF2, vRsoP-WM2 and vRsoP-WR2 genomes and a selection of 26 genomes of closely related *R. solanacearum* and *R. pseudosolanacearum* phages from other sources and continents. The heatmap incorporates intergenomic similarity values (top-right half) and alignment indicators (bottom-left half): aligned fraction of genome in row, genome length ratio and aligned fraction of genome in column. The black lines separate the predicted genera and the gray dashed lines separate the species. The geographical origin of the phages is indicated by the following colours: Africa (orange), Asia (green), Europe (blue) and America (purple).

**Figure 6:**
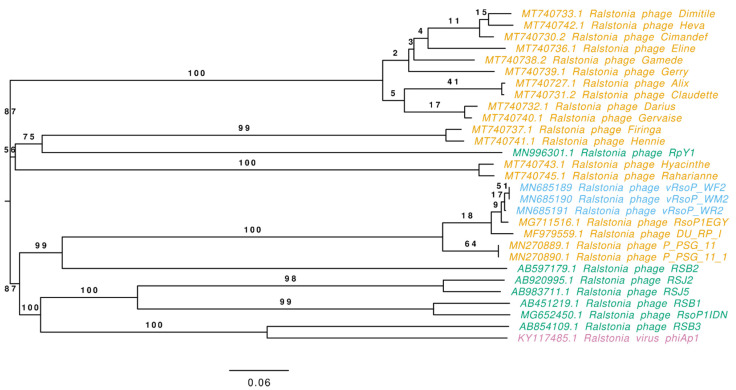
Phylogenetic tree based on genomic distances of European water phages vRsoP-WF2, vRsoP-WM2 and vRsoP-WR2 with a selection of closely related *R. solanacearum* and *R. pseudosolanacearum* phages from other sources and continents using VICTOR to calculate intergenomic distances. The geographical origin of the phages is indicated by the following colours: Africa (orange), Asia (green), Europe (blue) and America (purple).

**Figure 7:**
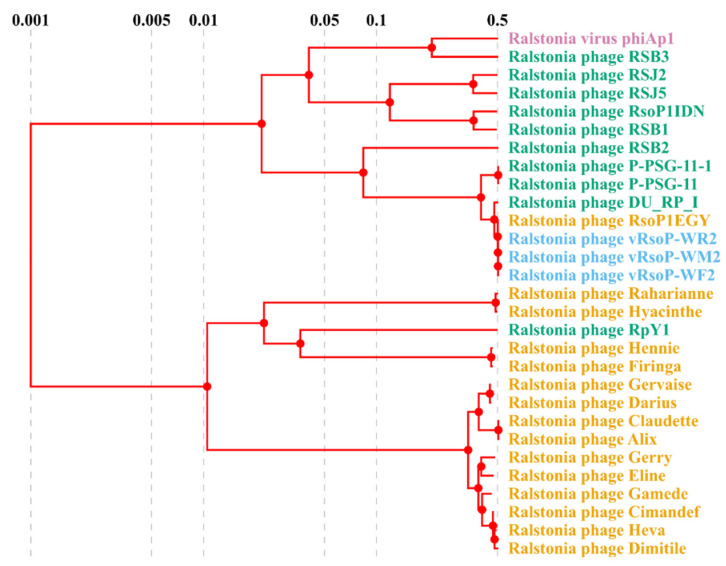
Phylogenetic proteomic tree of European water phages vRsoP-WF2, vRsoP-WM2 and vRsoP-WR2 with a selection of closely related *R. solanacearum* and *R. pseudosolanacearum* phages from other sources and continents using the ViPTree. The geographical origin of the phages is indicated by the following colours: Africa (orange), Asia (green), Europe (blue) and America (purple).

**Figure 8:**
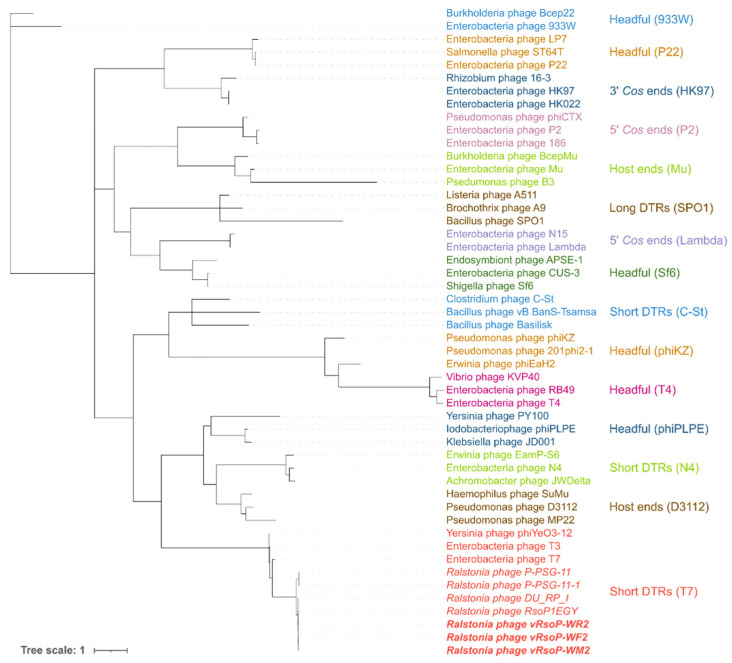
Phylogenetic tree of phage terminase large subunit (terL) protein of European water phages vRsoP-WF2, vRsoP-WM2 and vRsoP-WR2 (in bold and italics) with a selection of closely related *R. solanacearum* (in italics) and 50 reference phages. The name of each phage or prophage is indicated at each terminal node while the type of DNA packaging of each group, as in [57], is shown coloured. The lower part of the figure, in red, shows that the European *R. solanacearum* phages and their closely related phages cluster with the T7-like reference phages that use direct terminal repeats (DTR) as a packaging mechanism.

The nucleotide and protein sequences of the three European water phages were used to classify the phages. Phylogenetic analysis of the whole genomes of these three water phages and a selection of their 26 closest *Autographiviridae* (formerly *Podoviridae*) viruses infecting *R. solanacearum* and/or the closely related species *R. pseudosolanacearum* using VIRIDIC, allowed the classification of the 29 analysed phages into 20 species and 11 genera (Figure 5). The phylogenetic tree based on genomic distances of phages using VICTOR showed two clusters (Figure 6). In the upper cluster, all the African phages of *R. pseudosolanacearum* from plant, soil and water samples from Mauritius and Reunion islands [49] and the Asian phage RpY1 from Korean soil [52] were grouped. In the lower cluster were most of the Asian *R. pseudosolanacearum* phages and the only *R. solanacearum* American phage from Brazilian soil; in a subcluster were the three European *R. solanacearum* water phages (intergenomic similarity > 99%) together with the Egyptian *R. solanacearum* soil RsoP1EGY phage (intergenomic similarity ranging from 99 to 98.5%, depending on the phage), and more distant phages were located the Asian *Ralstonia* sp. DU_RP_I phage (intergenomic similarity about 95%), the African PSG-11 phage (intergenomic similarity >80%), and its heat adapted PSG-11-1 phage, isolated from water sources in Kenya [46]. Thus, VIRIDIC and VICTOR grouped vRsoP-WF2, vRsoP-WM2 and vRsoP-WR2 into the same new species, together with the phage RsoP1EGY (MG711516.1), isolated in Egypt more than a decade after the three European phages, whose activity against *R. solanacearum* was patented in 2017 because of their novelty and biocontrol efficiency [24]. This novel species shared the genus with phages DU_RP_I (MF979559.1), P-PSG-11 (MN270889.1) and P-PSG-11-1 (MN270890.1) (Figure 6). The phylogenetic analysis based on the viral proteomic tree using ViPTree (Figure 7) yielded a similar tree to that obtained using VICTOR, hence providing additional support to taxonomic results with VICTOR and VIRIDIC. Thus, the three phages belong to the same novel species that was further classified within the genus *Gyeongsanvirus* of the *Autographiviridae* family (Figure 5, Figure 6 and Figure 7). In order to determine the DNA packaging mechanism of the three European *R. solanacearum* phages, a phylogenetic tree was generated for the *terL* gene encoding for the large terminase subunit. The results revealed that the three phages clustered with each other and with the most closely related phages, which also supports the results of the phylogenetic analysis with the complete genomes (Figure 8). In addition, vRsoP-WF2, vRsoP-WM2 and vRsoP-WR2 clustered with T7-like phages, which use a packaging mechanism belonging to short DTR (Figure 8). Phage termini were sequenced by primer walking, allowing us to identify DTRs.

## 4. Discussion

The first European specific lytic phages successfully used to control the Gram-negative bacterium *R. solanacearum* are vRsoP-WF2, vRsoP-WM2 and vRsoP-WR2 [5,24,25,26]. Thus, biocontrol based on the activity of these waterborne phages can be considered a new strategy in bacterial wilt integrated management programs to be applied in host plants and irrigation water [5,24,25,26]. However, more knowledge on their genome and biology is required for a better understanding of their biocontrol abilities. 

Virions of vRsoP-WF2, vRsoP-WM2 and vRsoP-WR2 showed similar morphology determined by transmission electron microscopy, icosahedral heads of about 50 nm with short non-contractile tails, thus most likely belonging to the *Autographiviridae* family. These results confirmed previous data [5,24,25,26] and, from a more detailed study performed in this work, some variability among these phages was revealed, mainly in the tail length, which ranged from 19 to 26 nm, being longer for vRsoP-WF2. When comparing the three phages with the closely related *R. solanacearum* phage RsoP1EGY subsequently isolated from soil in Egypt, whose capsid diameter is around 60 nm and tail length is about 15 nm [47], the heads of the European phages were smaller and their tails longer. 

The DNA of the three phages was sequenced and the genomic information obtained was analysed in detail in this study. The average genome size of the European phages was 40,564 bp, with an average GC% of 59%. This GC contents is lower than that of *R. solanacearum* (66–67%) [16], as described for other phages in relation to their bacterial hosts [70]. It has been suggested that these differences between the host and its intracellular pathogen could be due to the competition for metabolic resources [71]. Phage genomes were very closely related among them (>99%), although with minor insertions and deletions, except in vRsoP-WR2, which also possesses a large insertion (468–469 bp), absent in the other two phages. Furthermore, they all share genomic organization except for an extra ORF (ORF 17) in vRsoP-WR2 in the large insertion, that was predicted to encode for an extra endonuclease. It is interesting to mention that vRsoP-WR2 seemed to have slightly higher *R. solanacearum* biocontrol efficiency *in planta* when single phages were applied [5,24,25,26]. 

These European phages showed high homology and shared similar genomic organization with some closely related *R. solanacearum* or *Ralstonia* sp. phages, RsoP1EGY, DU RP I, P-PSG-11 and P-PSG-11-1 [46,47]. In fact, vRsoP-WR2 shared with RsoP1EGY a large insertion of similar size [47]. However, it should be noted that, despite the fact that at sequence level the African RsoP1EGY phage exhibited greater homology with the European phages, the genomic organization was more similar to that of the Asian phage DU_RP_I.

The genomes of the European *R. solanacearum* phages contain 52 or 53 ORFs with only 22 or 23 with hypothetical functions and five transmembrane domains. Thus, most of their ORFs were hypothetical proteins lacking homology to known sequences. Among annotated ORFs, some shared homology with those predicted in other close *Ralstonia* phages mostly involved in conserved functions related with their lytic cycle (DNA replication and regulation, virion structure and host lysis), while others exhibited low similarity to known proteins. Genome analysis also revealed that they did not carry ORFs related with virulence and antibiotic resistance, which is preferred for their use as safe biocontrol agents.

The prediction of DNA and RNA polymerases in the genomes of the three European phages confirmed their classification within the *Autographiviridae* family [72] and hence, their lytic lifestyle. Like other lytic phages, they have evolved strategies to enter and lyse the host cell by the expression of enzymes which allow them to overcome the different barriers of the host cell, such as lysins and EPS depolymerases, that have been extensively studied in other phages for their potential applications in the control of bacterial pathogens, even in agriculture [73]. 

Lytic phages can weaken the host cell wall by means of lysins. These enzymes are expressed towards the end of the phage replication to degrade the host cell wall and allow the release of the viral progeny. For most tailed phages, clear plaques are considered an indicator of a phage being lytic. The plaque morphology of each of the three phages on lawns of *R. solanacearum* was clear halos due to their lytic activity [5,24] and, in this study, this activity was observed on four different bacterial culture media and with two distinct inoculation methods. This lytic activity was also detected based on clearing of *R. solanacearum* suspensions. Bioinformatic analysis of the phage genomes allowed the prediction of two putative lysins: (i) a peptidoglycan recognition protein (ORF 20 in vRsoP-WF2 and vRsoP-WM2, and ORF 21 in vRsoP-WR2) with a conserved amidase domain and high identity (89–100%) only with the closest *Ralstonia* phages, and (ii) a peptidoglycan lytic exotransglycosylase with a lysozyme-like domain found in glycosidases and transglycosylases (ORF 47 in vRsoP-WF2 and vRsoP-WM2, and ORF 48 in vRsoP-WR2). Most of the endolysins reported so far are lytic transglycosylases [74]. Two putative virion-associated peptidoglycan hydrolases have been recently reported in the American *R. solanacearum* phiAP1 phage, being proposed to act as exolysins, which locally pierce peptidoglycan to eject the phage DNA into the host, in the first steps of the infection [48], as already reported in other phages of the *Autographiviridae* family, and like one of the lysins expressed by these European phages (ORF 47 and 48) but, with a poorly conserved region when compared with the phiAP1 phage. The two identified lysins in the three European phages will be further characterized to better determine their contribution to the biological control of the bacterial wilt disease.

For most phages infecting Gram-negative bacteria, cell lysis involves the expression of holins/endolysins/spanins for the disruption of the inner membrane, peptidoglycan and outer membrane, respectively. Neither holins nor spanins were identified as such in the European phages, nor in the closest *Ralstonia* phages. However, when searching for domains of transmembrane helices in proteins using TMHMM software, 5 ORFs were found that are predicted to contain these transmembrane domains, and whose presence has been described in holins and spanins [67,68].

EPS is one of the most important virulence determinants of *R. solanacearum* [75,76], causing bacterial wilt by blocking water flow in the host xylem vessels and protecting the pathogen from plant antimicrobial defenses [77,78]. Most short-tail phages able to infect bacteria that produce EPS encode for depolymerases that can degrade bacterial EPS to gain access to the host surface receptors [79,80] and also make the pathogen more susceptible to plant defenses [81]. Given the key role of EPS in *R. solanacearum* pathogenesis and the potential contribution and/or use of phage-depolymerases for biocontrol of bacterial wilt, this potential activity that can be detected by visualization of phage plaques surrounded by expanding translucent halos was further investigated [80]. In this study, it was found that the three European phages produced turbid halos around clear plaques growing over time, indicative of depolymerase-containing phages [82]. Furthermore, these growing halos were more easily observed in the two bacterial culture media with glucose (YPGA and CPGA), where *R. solanacearum* produces more fluidal colonies because of an increased EPS production.

Most phage depolymerases are associated with phage structural components, such as tail fibers [80]. In this study, ORF 48 in vRsoP-WF2 and vRsoP-WM2, and ORF 49 in vRsoP-WR2 were identified, which encoded for a putative tail fiber with EPS depolymerase activity. Phage tail fiber proteins contain three domains: the N-terminal, related to the binding of phage tails, the central domain for host recognition and depolymerase activity, and the C-terminal domain responsible for protein trimerization and/or host recognition [83]. The N-terminal and C-terminal domains have been reported to be conserved among phages of the same group, while the central domain, involved in host-specificity, has been described as highly variable and can be changed to modulate the phage host range or to adapt to novel environments [83]. These two conserved domains have been reported to be present in other phage tail spike proteins involved in EPS degradation [69]. Bioinformatic analysis of ORFs 48 and 49 coding for tail fiber proteins in the three European phages allowed the identification of the domain IPR011050, associated with pectin lyase activity, which is involved in the cleavage of glycoside bonds in capsular polysaccharides and essential for initiation of phage infection. A short tail fiber was recently annotated in the ORF 43 of the *R. solanacearum* phage phiAP1 with a RmLc-like cupin domain, present in sugar isomerases with EPS depolymerase activity but, no experimental validation was performed [48]. No significant similarity was found between this ORF and ORFs 48 and 49 of the European *R. solanacearum* phages. However, these ORFs have two domains equivalent to those in the N-terminal and the central regions of the ORF 42 of the *K. pneumoniae* phage SH-KP152226 [69]. In the central region (residues 288~628) of ORF 42, a pectin lyase fold, involved in bacterial EPS degradation, was identified. The protein encoded by ORF 42 of phage SH-KP152226 was recently cloned, expressed and experimentally confirmed as a depolymerase [69]. Therefore, in our work, this type of depolymerase in *R. solanacearum* phages was identified for the first time. Since they could confer competitive advantages to these phages by allowing them to penetrate *R. solanacearum* biofilms and favouring host cell infection in some niches, they will be further explored to elucidate their role in biocontrol, as well as their potential therapeutic use. Finally, the potential combined use of these phages with their lytic and/or depolymerizing enzymes will be further explored to optimize their biocontrol activity.

As previously described, terL protein sequence can be useful for determining phylogenetic relationships between phages, as well as for predicting phage DNA packaging strategy [56,57,84]. The phylogenetic tree of terL with vRsoP-WF2, vRsoP-WM2 and vRsoP-WR2 was consistent with the one obtained using the complete genomes. Moreover, the three European phages clustered with T7-like phages, which use a sequence-specific packaging mechanism, short DTR [84]. To confirm this prediction, we experimentally identified and located the termini of the three phages by Sanger sequencing, showing that their DNA packaging mechanism is based on DTR. This was due to the fact that the fragments generated during the preparation of the Nextera libraries used to sequence the three phages made it difficult to determine the phage ends, which is consistent with previous publications [85].

The three European *R. solanacearum* phages showed high similarity among their genomes and were taxonomically classified within the same species. The latter could be indicative of their common origin, *R. solanacearum* contaminated river water samples in Spain, although from three different regions geographically distant from each other, and different years of isolation. Their genomes exhibited low similarity to other phages infecting *Ralstonia* spp., and mostly those from Asia were the first *Ralstonia* phages reported as well as those from Africa, in both cases mainly isolated against *R. pseudosolanacearum,* the species more prevalent under field conditions in these two continents [4,49,50], except for one Asian *Ralstonia* sp. phage, DU_RP_I and three African phages recently reported, RsoP1EGY, P-PSG-11 and P-PSG-11-1 [46,47]. Among them, the most closely related to the three European phages was RsoP1EGY, up until now not fully classified but, in agreement with the present phylogenetic analysis, belonging to the same and novel species as vRsoP-WF2, vRsoP-WM2 and vRsoP-WR2. Since the three European *R. solanacearum* phages were isolated from water sources in 2001, 2003 and 2004, RsoP1EGY was obtained from soil in Egypt in 2017, and the closest Asian phage DU_RP_I was described in 2017 (sequence published in 2017), the question arising is how can it be explained that phages from three different continents share high similarities. As previously suggested for dsDNA tailed phages [86], these closely related phages able to infect *Ralstonia* spp. probably share a common ancestry.

## 5. Conclusions

In the present study, the morphological and genomic characterization of the first European phages with biocontrol efficacy against *R. solanacearum* in host plants and irrigation water were described. The results have allowed their taxonomic classification as members of a novel species of the genus *Gyeongsanvirus* within the *Autographiviridae* family. The experimental and bioinformatic analysis of vRsoP-WF2, vRsoP-WM2 and vRsoP-WR2 have confirmed their suitability as safe biocontrol agents and provided new information on their lytic and EPS depolymerase proteins, which better explain their capacity for the biocontrol of *R. solanacearum.* The new data also will allow the improvement of the understanding of the interactions among these phages and their pathogenic host. 

## Figures and Tables

**Table 1:** Characteristics of the genomes of the three European *R. solanacearum* phages.

Phage	Length	GC-Contents	Annotated Proteins	GenBank Accession Number
vRsoP-WF2	40,690 bp	59.07%	52	MN685189
vRsoP-WM2	40,688 bp	59.10%	52	MN685190
vRsoP-WR2	41,158 bp	59.04%	53	MN685191

**Table 2:** Characteristics of the genomes of the European *R. solanacearum* phages.

ORF Number			Predicted Function
vRsoP-WF2	vRsoP-WM2	vRsoP-WR2	
1	1	1	
2	2	2	
3	3	3	
4	4	4	
5	5	5	
6	6	6	
7	7	7	
8	8	8	
9	9	9	
10	10	10	
11	11	11	
12	12	12	
13	13	13	
14	14	14	Phage integrase, tyrosine recombinase
15	15	15	Phage HNH homing endonuclease
16	16	16	Phage DNA-directed RNA polymerase
-	-	17	Phage restriction endonuclease
17	17	18	
18	18	19	Phage single-stranded DNA-binding protein
19	19	20	Phage endonuclease I
20	20	21	Phage endolysin
21	21	22	
22	22	23	Phage restriction endonuclease
23	23	24	Phage primase/helicase protein
24	24	25	
25	25	26	
26	26	27	Phage DNA-directed DNA polymerase
27	27	28	
28	28	29	
29	29	30	
30	30	31	Phage exonuclease
31	31	32	
32	32	33	
33	33	34	
34	34	35	
35	35	36	Phage collar, head-to-tail connector protein
36	36	37	Phage capsid assembly protein
37	37	38	
38	38	39	Phage capsid and scaffold protein
39	39	40	Phage endonuclease VII
40	40	41	Phage major capsid protein
41	41	42	
42	42	43	Phage tail tubular protein
43	43	44	Phage non-contractile tail tubular protein
44	44	45	Phage protein, probable scaffold protein
45	45	46	
46	46	47	
47	47	48	Phage DNA ejectosome component, peptidoglycan lytic exotransglycosylase
48	48	49	Phage tail fiber protein, pectin-lyase fold
49	49	50	
50	50	51	Phage terminase small subunit
51	51	52	
52	52	53	Phage terminase large subunit

## Data Availability

Sequences of three phages were available in GenBank with accession numbers MN685189 (vRsoP-WF2), MN685190 (vRsoP-WM2) and MN685191 (vRsoP-WR2).

## Supplementary Materials

The following are available online at https://www.mdpi.com/article/10.3390/v13122539/s1, Table S1: ORFs detailed information of the European R. solanacearum phages vRsoP-WF2, vRsoP-WM2 and vRsoP-WR2. Table S2: Data from the phages or prophages terminase large subunit proteins used for the DNA packaging method prediction (Figure 8).
